# Supplementary material for: Charge density waves and Fermi surface reconstruction in the clean overdoped cuprate superconductor Tl2Ba2CuO6+δ
Source: Nat Commun. 2022 Jan 28;13:570. doi: 10.1038/s41467-022-28124-y (PMC8799688; doi:10.1038/s41467-022-28124-y)
Supplement: Supplementary file 1 — Supplementary Information [file 41467_2022_28124_MOESM1_ESM.pdf]

Charge density waves and Fermi-surface reconstruction in the clean overdoped cuprate superconductor $Tl_2Ba_2CuO_{6+\delta}$

Supplementary Information

C. C. Tam,[1,2] M. Zhu,[1] J. Ayres,[1] K. Kummer,[3] F. Yakhou-Harris,[3] J. R. Cooper,[4] A. Carrington,[1] and S. M. Hayden[1]

[1]*H. H. Wills Physics Laboratory, University of Bristol, Bristol BS8 1TL, United Kingdom.*
[2]*Diamond Light Source, Harwell Campus, Didcot OX11 0DE, United Kingdom.*
[3]*ESRF, The European Synchrotron, 71 Avenue des Martyrs, CS40220, 38043 Grenoble Cedex 9, France.*
[4]*Department of Physics, University of Cambridge, Madingley Road, Cambridge, CB3 0HE, United Kingdom.*

SUPPLEMENTARY NOTE 1: ACCESSIBLE RECIPROCAL SPACE

With soft x-rays, ($\sim 930\,\mathrm{eV}$ at Cu-L_3), the accessible reciprocal space is limited due to the large wavelength of the x-rays compared to the unit cell. The detector arm of the ID32 spectrometer can move in the range $50° < 2\theta < 150°$, where 2θ is the scattering angle of the x-rays. The sample rotation Ω is limited by the angle between in the incoming (α) and outgoing (β) x-rays and the surface of the sample (See Fig. 1a). We are able to work with $\alpha, \beta \gtrsim 9°$. Thus, the accessible region of the $(H, 0, L)$ is shown by the green region of Fig. 1b. For $H = -0.31$, we can access $2 \lesssim L \lesssim 2.8$.

For our measurements, we chose $L = 2.5$ to give us a region of H of either side of $H = -\delta$. For comparison, Table 1 summarises the measurement conditions for some other RIXS studies on cuprates where CDWs have been observed for various L values. This compilation is consistent with the CDW scattering being spread out in L and peaked at certain positions.

Tl2201 has the same body-centered-tetragonal crystal structure ($I4/mmm$) as LSCO which has the strongest CDW peaks near L = integer + 1/2 positions [1]. Hg1201 has a primitive structure ($P4/mmm$) with the strongest CDW peaks being found at $L = 1$ [2].

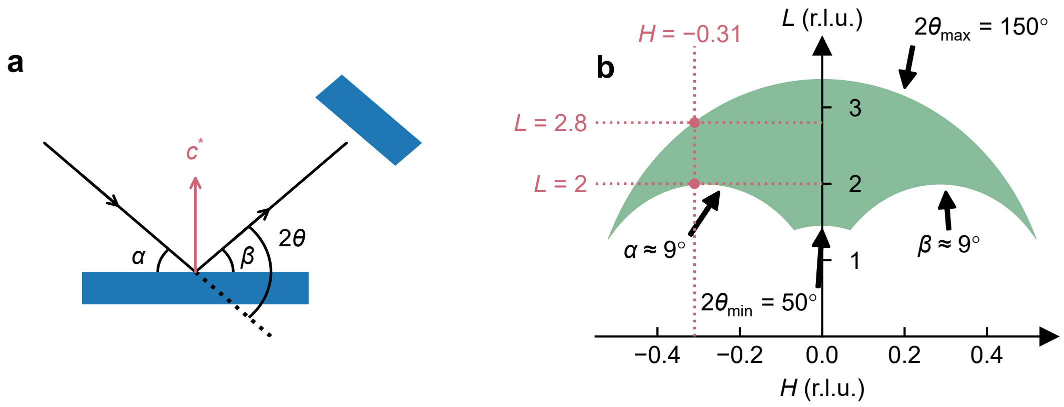

Supplementary Fig. 1: **a.** Scattering geometry. **b.** Accessible k-space at Cu-L, given restrictions on Ω and 2θ.

Material	Reference	Doping p	δ (r.l.u.)	2θ (°)	c (Å)	L at δ (r.l.u.)
LSCO	[3]	0.12	0.23	120	13.28	1.5
Bi2201	[4]	0.13	0.26	154	24.69	3.2
Bi2212	[5]	0.08	0.3	149.5	30.84	3.7
Hg1201	[6]	0.09	0.28	160	9.53	1.25

Supplementary Table 1: Measurement conditions of various fixed resonant Cu-L studies of the CDW in cuprates with fixed 2θ.

SUPPLEMENTARY NOTE 2: PHONON SPECTRA AND FITTING

The fitting model of the RIXS spectra was comprised of two resolution limited Gaussians, accounting for elastic scattering and a phonon, a damped harmonic oscillator (DHO) to fit spin excitations and finally a linear background, all fitted in the range $[-100, 300]$ meV. The damping parameter of the DHO was fixed to half of the DHO frequency and the whole function was numerically convolved over $\pm 4\sigma$ with a resolution limited Gaussian. The DHO model used had the form

$$S(\omega; \omega_0, \gamma, I) = \frac{1}{1 - \exp(-\hbar\omega/k_B T)} \frac{I\gamma\omega_0^2\omega}{(\omega - \omega_0^2)^2 + (\omega\gamma)^2}, \quad (1)$$

where ω_0 is the DHO frequency, γ is the damping parameter and I is the intensity.

RIXS spectra collected for our samples of Tl2201 had relatively strong elastic scattering. This makes the phonon less pronounced than in some other RIXS studies e.g. [3] where the phonon peak is stronger than the elastic peak at the zone edge. Thus phonon fitting with acceptably small error bars could only be done on a small subset of measurements we took. The data presented in the main text in Fig. 2 was counted for about 17 minutes per spectrum.

The spectra for the $p = 0.25$ composition at $T_c = 45\,\mathrm{K}$ and the $p = 0.23$ composition taken at $T_c = 56\,\mathrm{K}$ are plotted in Fig. 2. Note the $p = 0.23$ measurements were counted for about 4 minutes per spectrum, resulting in noticeably worse statistics. The resulting phonon dispersions are plotted in Fig. 3. The statistics and range of H for the $p = 0.23$ data means that little can be said about phonon softening. For the $p = 0.25$ data, the mean phonon energy for $|H| > 0.31 = \delta$ is lower than that in the region $|H| \approx 0.15$ which is consistent with a softening of $\sim 10\%$. However, this result is statistically very weak. Thus, we conclude that our data is consistent with a softening of up to 10%, but our experiment is not able to say definitively whether there is phonon softening or not and further work is required to clarify this issue.

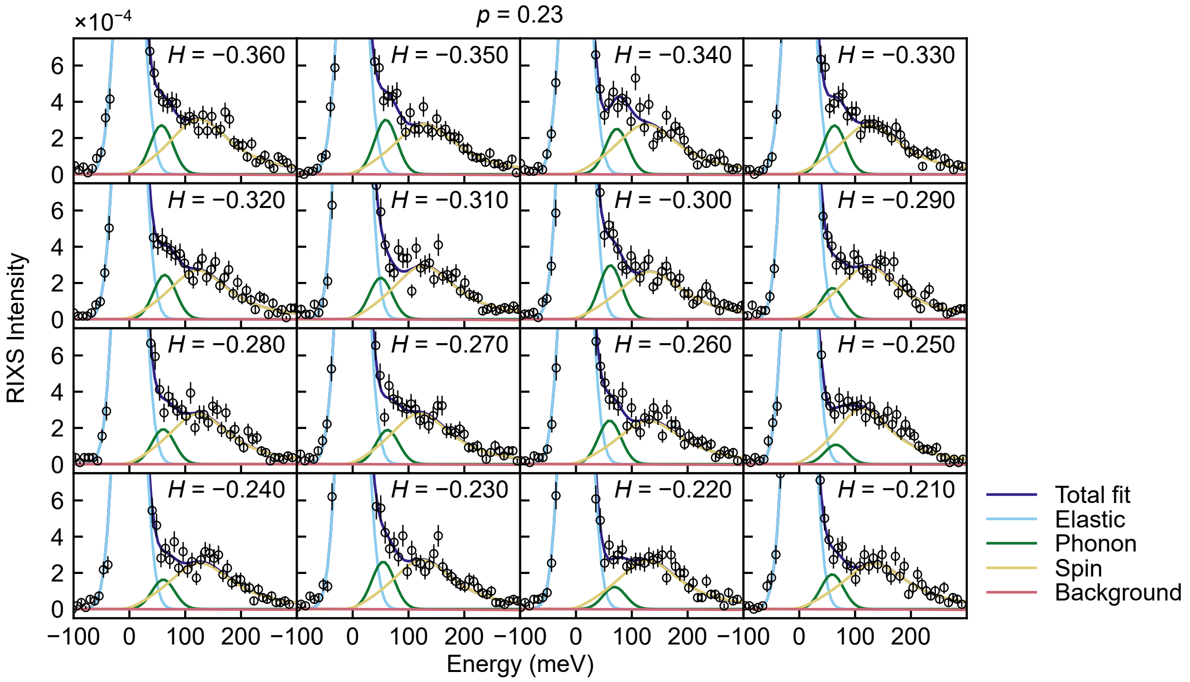

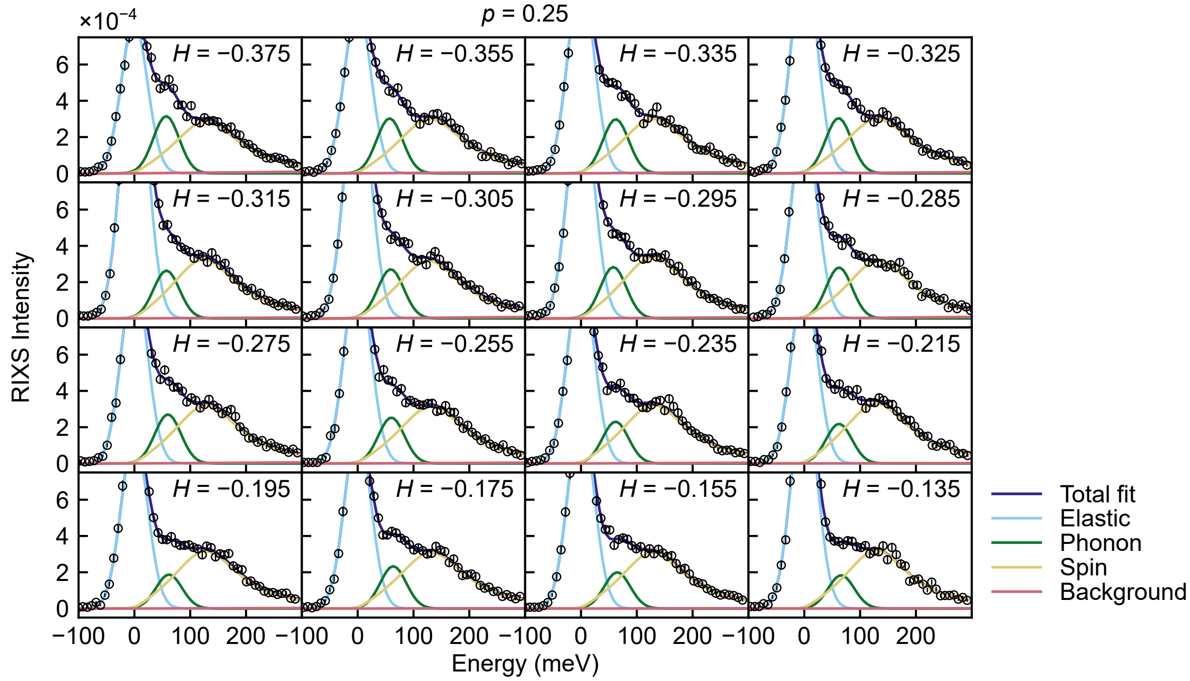

Supplementary Fig. 2: Low-energy region of RIXS spectra for $p = 0.23$ (top) and $p = 0.25$ (bottom), with components of the fit plotted.

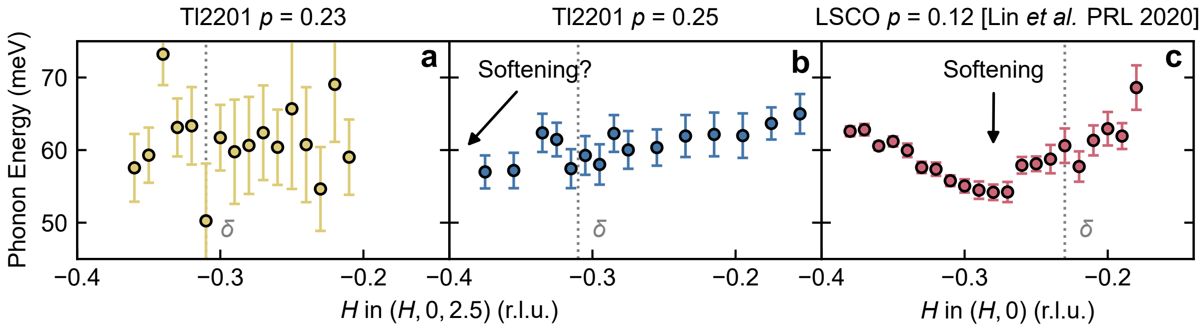

Supplementary Fig. 3: Bond stretching phonons in single layered cuprates. The gray dotted line marks the position of δ in each material. Phonon energies from fitting for **a.** $p = 0.23$ and **b.** $p = 0.25$. **c.** LSCO $p = 0.12$ from Ref. [3].

SUPPLEMENTARY NOTE 3: SEARCH FOR HIGH-ENERGY CHARGE FLUCTUATIONS

Boschini *et al.* [7] have recently observed an inelastic ring-like feature in the (H, K) plane of Bi2212, which was interpreted as evidence for dynamical charge fluctuations. With the integral range used by Boschini *et al.* of $[500, 900]$ meV, we found no evidence for a feature like this in our ζ scans (see Fig. 4).

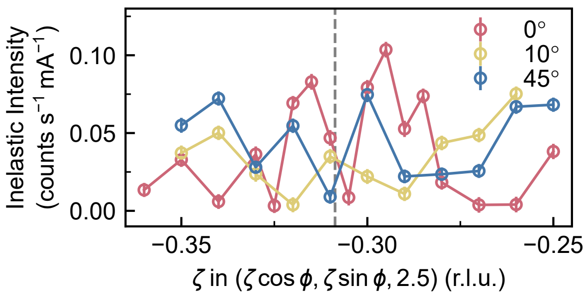

Supplementary Fig. 4: $[500, 900]$ meV integral of RIXS scans of $p = 0.23$. Dotted line marks $\delta \approx -0.31$.

SUPPLEMENTARY NOTE 4: COMPARISON TO OTHER CUPRATES

It is interesting to compare the H-integrated CDW intensity to other cuprates. We compared to LSCO ($p = 0.12$) [8], measured at $T_c = 30$ K with a fixed $L = 1.5$. The area of the spectra in the range $[1000, 3000]$ meV is used to normalise between materials. The elastic intensity is integrated in the range $[-45, 45]$ meV to avoid the strong phonon in LSCO. The RIXS spectra for Tl2201 and LSCO at their respective CDW positions are plotted in Fig. 5a. In Fig. 5b we plot the H-dependent elastic RIXS intensity. The integrated intensity and correlation lengths of the fits to this data are given in Table. 2. The H-integrated intensity of the CDW Bragg peaks in Tl2201 ($p = 0.23$) and LSCO ($p = 0.12$) have similar values.

Material	Doping p	H-Integrated CDW Intensity	ξ (Å)
Tl2201	0.25	$(6.6 \pm 0.5) \times 10^{-4}$	206 ± 15
Tl2201	0.23	$(1.8 \pm 0.1) \times 10^{-3}$	138 ± 9
LSCO [8]	0.12	$(2.2 \pm 0.1) \times 10^{-3}$	25.3 ± 1.3

Supplementary Table 2: H-Integrated CDW intensity I_{CDW} and correlation length of the CDW in Tl2201 and LSCO $p = 0.12$ [8].

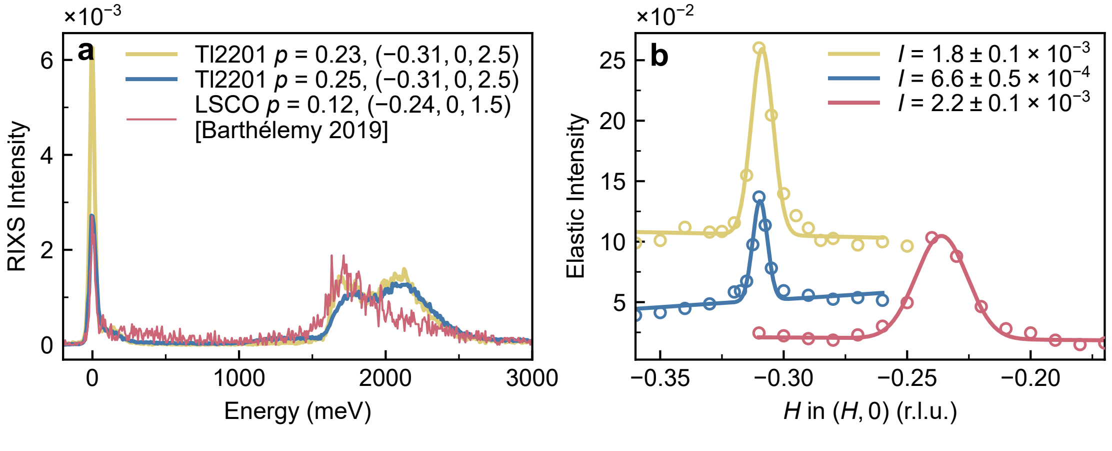

Supplementary Fig. 5: Comparison of CDW in Tl2201 to LSCO $p = 0.12$. **a.** RIXS spectra taken near the CDW position of Tl2201 $p = 0.23$ in yellow, Tl2201 $p = 0.25$ in blue and LSCO $p = 0.12$ [8] in red. Spectra have been normalised to the area of the dd excitations in $[1000, 3000]$ meV. **b.** H-dependent scans of the elastic RIXS intensity, taken as the integral in $[-45, 45]$ meV of Tl2201 and LSCO. All measurements are taken at T_c of the respective sample.

SUPPLEMENTARY NOTE 5: HALL COEFFICIENT CALCULATIONS

Calculations of the Hall coefficient in the weak-field limit were performed using a two dimensional approximation of the Fermi surface constructed from the ARPES derived tight-binding parameters, and reconstructed with a CDW potential $Q_{\mathrm{CDW}} = (2\pi/3, 0)$ and $Q_{\mathrm{CDW}} = (0, 2\pi/3)$ as as outlined in the Methods. Calculations were performed on a grid of 10^6 k-points. The weak-field expressions for longitudinal and Hall conductivities were calculated using the standard expressions

$$\sigma_{xx} = \frac{e^2}{2\pi^2} \int \left(-\frac{\partial f}{\partial \varepsilon}\right) \tau v_x^2 d^2k \tag{2}$$

$$\sigma_{xy} = \frac{e^3 B}{2\pi^2 \hbar^2} \int \left(\frac{\partial f}{\partial \varepsilon}\right) \tau^2 \left(v_x v_y \frac{\partial v_y}{\partial k_x} - v_x v_x \frac{\partial v_y}{\partial k_y}\right) d^2k. \tag{3}$$

Here v_x and v_y are the components of the velocity ($v_x = \hbar^{-1}\partial\varepsilon/\partial k_x$), f is the Fermi function, B is the magnetic field, $\varepsilon(k)$ is the quasiparticle energy and τ is the scattering lifetime which we took as constant. This assumption on τ is consistent with the low temperature limit of the behaviour of $\tau(T)$ derived from angle dependent magnetoresistance measurements [9], and so we would expect our results to apply in the low temperature limit. The results were only weakly dependent on the temperature in the Fermi function and we used $T = 20\,\mathrm{K}$ for the calculation (the size of T is chosen so that the finite k point spacing does not cause problems with the integral). The Hall number is finally calculated as $n_H = (B/e)\sigma_{xx}^2/\sigma_{xy}$, and is expressed in units of carriers per unit cell.

To model the experimental data we assumed that the CDW potential, V_0, increases linearly with decreasing doping p as shown in Fig. 6, being zero for $p > 0.255$ and saturating at $V_0 = 60\,\mathrm{meV}$ for $p = 0.1425$. Increasing V_0 causes the electron pocket to shrink and the Fermi velocities on this pocket to be reduced. σ_{xy} is the sum of the positive and negative contributions from the hole and electron pockets respectively, so a reduction in magnitude of the (negative) electron contribution causes σ_{xy} to increase and hence n_H to decrease. On the other hand, reducing p with fixed V_0 causes the electron pocket contribution to grow. Hence increasing V_0 while reducing p produces two opposing effects on the size of the electron and hole pockets and their contribution to n_H.

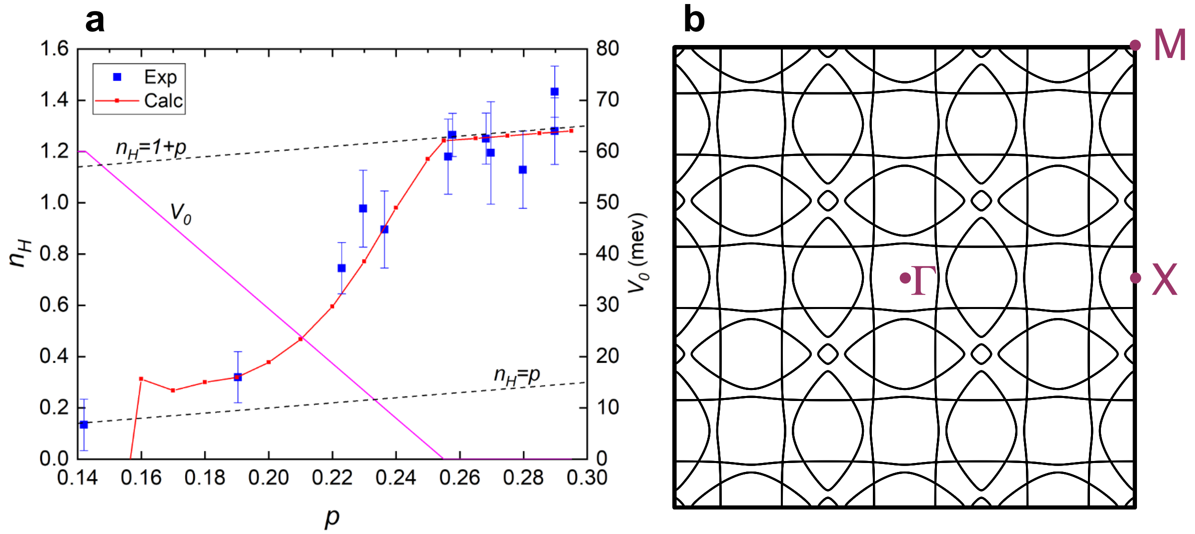

Supplementary Fig. 6: **a** Hall number n_H versus doping for Tl2201. Blue squares are experimental data from Ref. [10], and small red squares are the calculated values. The assumed variation of the CDW potential V_0 with doping p is shown with the magenta solid line. **b** Fermi surface reconstruction in Tl2201 ($p = 0.25$) calculated using the model described in the Methods Section for $V_0 = 10$ meV.

With our assumed p dependence of V_0, the calculated behaviour of n_H is in good agreement with the experimental data as shown in Fig. 6. For $p > 0.255$ $n_H \simeq (1 + p)$, consistent with the large Fermi surface found in DFT and quantum oscillation measurements. As the CDW begins to open, for $p < 0.255$, n_H falls towards values closer to $n_H = p$. For $p < 0.16$, n_H changes sign because the hole pocket disappears, however whether this occurs or not

depends on the gradient, dV_0/dp. We note that our calculation does not include any modelling of the pseudogap, which would be expected to reduce the contribution of the hole pocket for p inside the pseudogap regime ($p \lesssim 0.19$) and hence make a sign change of n_H more likely.

[1] T. P. Croft, C. Lester, M. S. Senn, A. Bombardi, and S. M. Hayden, Charge density wave fluctuations in $La_{2-x}Sr_xCuO_4$ and their competition with superconductivity, Phys. Rev. B **89**, 224513 (2014).

[2] W. Tabis, B. Yu, I. Bialo, M. Bluschke, T. Kolodziej, A. Kozlowski, E. Blackburn, K. Sen, E. M. Forgan, M. v. Zimmermann, Y. Tang, E. Weschke, B. Vignolle, M. Hepting, H. Gretarsson, R. Sutarto, F. He, M. L. Tacon, N. Barišić, G. Yu, and M. Greven, Synchrotron x-ray scattering study of charge-density-wave order in $HgBa_2CuO_{4+\delta}$, Phys. Rev. B **96**, 134510 (2017).

[3] J. Lin, H. Miao, D. Mazzone, G. Gu, A. Nag, A. Walters, M. García-Fernández, A. Barbour, J. Pelliciari, I. Jarrige, M. Oda, K. Kurosawa, N. Momono, K.-J. Zhou, V. Bisogni, X. Liu, and M. Dean, Strongly correlated charge density wave in $La_{2-x}Sr_xCuO_4$ evidenced by doping-dependent phonon anomaly, Phys. Rev. Lett. **124**, 207005 (2020).

[4] J. Li, A. Nag, J. Pelliciari, H. Robarts, A. Walters, M. Garcia-Fernandez, H. Eisaki, D. Song, H. Ding, S. Johnston, R. Comin, and K.-J. Zhou, Multiorbital charge-density wave excitations and concomitant phonon anomalies in $Bi_2Sr_2LaCuO_{6+\delta}$, Proc. Natl. Acad. Sci. U.S.A. **117**, 16219 (2020).

[5] L. Chaix, G. Ghiringhelli, Y. Y. Peng, M. Hashimoto, B. Moritz, K. Kummer, N. B. Brookes, Y. He, S. Chen, S. Ishida, Y. Yoshida, H. Eisaki, M. Salluzzo, L. Braicovich, Z.-X. Shen, T. P. Devereaux, and W.-S. Lee, Dispersive charge density wave excitations in $Bi_2Sr_2CaCu_2O_{8+\delta}$, Nat. Phys. **13**, 952 (2017).

[6] W. Tabis, Y. Li, M. L. Tacon, L. Braicovich, A. Kreyssig, M. Minola, G. Dellea, E. Weschke, M. J. Veit, M. Ramazanoglu, A. I. Goldman, T. Schmitt, G. Ghiringhelli, N. Barišić, M. K. Chan, C. J. Dorow, G. Yu, X. Zhao, B. Keimer, and M. Greven, Charge order and its connection with Fermi-liquid charge transport in a pristine high-T_c cuprate, Nat. Commun. **5**, 5875 (2014).

[7] F. Boschini, M. Minola, R. Sutarto, E. Schierle, M. Bluschke, S. Das, Y. Yang, M. Michiardi, Y. C. Shao, X. Feng, S. Ono, R. D. Zhong, J. A. Schneeloch, G. D. Gu, E. Weschke, F. He, Y. D. Chuang, B. Keimer, A. Damascelli, A. Frano, and E. H. da Silva Neto, Dynamic electron correlations with charge order wavelength along all directions in the copper oxide plane, Nat. Commun. **12**, 597 (2021).

[8] M. C. Barthélemy, *Studies of Charge Density Waves in Cuprate Superconductors*, Ph.D. thesis, University of Bristol (2019).

[9] M. Abdel-Jawad, M. P. Kennett, L. Balicas, A. Carrington, A. P. Mackenzie, R. H. McKenzie, and N. E. Hussey, Anisotropic scattering and anomalous normal-state transport in a high-temperature superconductor, Nat. Phys. **2**, 821 (2006).

[10] C. Putzke, S. Benhabib, W. Tabis, J. Ayres, Z. Wang, L. Malone, S. Licciardello, J. Lu, T. Kondo, T. Takeuchi, N. E. Hussey, J. R. Cooper, and A. Carrington, Reduced Hall carrier density in the overdoped strange metal regime of cuprate superconductors, Nat. Phys. **17**, 826 (2021).
