# Supplementary material for: Charge density waves and Fermi surface reconstruction in the clean overdoped cuprate superconductor Tl2Ba2CuO6+δ
Source: Nat Commun. 2022 Jan 28;13:570. doi: 10.1038/s41467-022-28124-y (PMC8799688; doi:10.1038/s41467-022-28124-y)
Supplement: Supplementary file 2 — Peer Review File [file 41467_2022_28124_MOESM2_ESM.pdf]

nature portfolio

Peer Review File

Charge density waves and Fermi-surface reconstruction in the clean overdoped cuprate superconductor Tl2Ba2CuO6+δ

Open Access This file is licensed under a Creative Commons Attribution 4.0 International License, which permits use, sharing, adaptation, distribution and reproduction in any medium or format, as long as you give appropriate credit to the original author(s) and the source, provide a link to the Creative Commons license, and indicate if changes were made. In the cases where the authors are anonymous, such as is the case for the reports of anonymous peer reviewers, author attribution should be to 'Anonymous Referee' followed by a clear attribution to the source work. The images or other third party material in this file are included in the article's Creative Commons license, unless indicated otherwise in a credit line to the material. If material is not included in the article's Creative Commons license and your intended use is not permitted by statutory regulation or exceeds the permitted use, you will need to obtain permission directly from the copyright holder. To view a copy of this license, visit http://creativecommons.org/licenses/by/4.0/.

REVIEWER COMMENTS

Reviewer #1 (Remarks to the Author):

In this paper, the authors Tam et al. present a comprehensive study of the charge density wave (CDW) order in overdoped $Tl_2Ba_2CuO_{6+\delta}$ (Tl2201) using resonant inelastic x-ray scattering at the Cu L3 edge. This cuprate compound is profoundly interesting because it is a structurally simple system whose doping level can be controlled from optimal to overdoped by varying the oxygen δ. Presumably the disorder level in these compounds is lower than in other cuprate families, certainly the Bi-based compounds which are full of diffraction peaks.

The results can be summarized as follows:
-For two doping values (p= 0.23 and 0.25) they see an elastic peak with q-vector near the same reciprocal lattice position that has been observed in other cuprates
-the peak seems to resonate only at the Cu L3 edge
-the peak grows as a function of decreasing temperature until the superconducting transition takes place, below which the peak intensity drops
-the in-plane correlation length (20 nm) of the peak is much higher than in other cuprates
-for a higher doping value of p=0.28, they observe no such elastic peak.

From these data, the authors conclude that there is long-range CDW order in this compound, in analogy to that observed in underdoped cuprates. This is a very important observation for the following reasons:
-although some claims some been made of CDW in the overdoped regime, it is still not clear where in the phase diagram the CDW phase ends. The fact that it is largely contained within the underdoped region has lead the community to associate this to the pseudogap phenomenon and other such puzzles of the underdoped regime. The previous result on Bi-2201 [Ref 26] is not very convincing because it has a very odd temperature dependence, more likely associated to a structural peak. In $La_{2-x}Sr_xCuO_4$, CDW order has been observed [ref 25], and this is more compelling to be attributed to an electronic state that is relevant to superconductivity. Other works have shown dynamical fluctuations (phonons or electronic) which may be a precursor to CDW that does not form. The work in this manuscript adds an important clue to this puzzle, which shows that in systems with low disorder, the CDW does prevail beyond the underdoped regime.
-this answers the question of whether a putative quantum critical point exists at the end of the CDW phase. It does not.
-Yet, the CDW observed here still competes with superconductivity (SC), which means that the CDWS-SC relation still holds even in the absence of the pseudogap.

Furthermore, the authors make an interesting connection between the CDW and the change in the low-temperature, high-field Hall number which points to a Fermi surface reconstruction. The CDW q-vector's doping dependence in Tl2201 does not fit any of the trends observed in other cuprates. The authors attempt to marry these findings with known properties of the Fermi surface of Tl2201. While these ideas are solid, more experimental work will be needed to confirm this picture.

The paper is well written and contains a very important piece of this puzzle, and should be published eventually in Nature Communications. However, there are some fundamental questions I would like the authors to address, as well as some extra data that is needed, as follows.

-The authors do not see the 60-meV phonon softening, which seems to happen in many other cuprates and has been associated with electron-phonon coupling. This is a crucially important clue here in the sense that if they do not see the softening, then the e-p coupling here might be weaker than in the underdoped case. Since this is so important, the authors should show how they extracted the phonon energy, and show more details of this analysis in the supplementary information. The resolution 48meV is comparable to that of other works, so the information of whether the phonon

disperses is already in their data. It should be presented thoroughly in this paper, for both samples that show a CDW.

-Recently an inelastic intensity feature was observed across the whole qx-qy plane (https://www.nature.com/articles/s41467-020-20824-7), and the authors have performed Q-RIXS maps across different azimuthal directions. In the current paper, the authors only show the elastic intensity as a function of azimuth, but they have the whole inelastic data to analyze. The authors should discuss if this inelastic feature seen in Bi2212 is observed in Tl2201.

Reviewer #2 (Remarks to the Author):

Tam et al., report Cu L3 edge RIXS measurements of overdoped Tl2Ba2CUO6+delta. They discover a previously unseen Bragg peak which they assign to charge density wave (CDW) correlations. The research delivers a clear new piece of information in a topical and interesting area. Indeed, there is widespread long-lasting interest in CDWs in cuprates, both as a fundamental ordering tendency in strongly correlated quantum materials and as regarding its relationship to high temperature superconductivity. The presentation of the manuscript is overall, very good. I have no serious critical remarks, just a couple of questions, which I list below. I consider the paper suitable for Nature Communications and suggest that it is accepted after the comments below have been considered by the authors.

There is some chance that other referees might comment that the results in Tl2Ba2CUO6+delta have some similarity to those in LSCO and BSCCO. It is true that there is some similarity in phenomenology, but I nonetheless feel that the results are interesting enough for Nature Communications, because it is important to identify phenomena that are shared among all cuprates. An example is the substantial interest in the CDW in underdoped BSCCO despite the substantial similarity of this to YBCO and, to a lesser extent, La-based cuprates. Speaking frankly, similarity between different cuprates is applied very inconsistently in review decisions and it often used by more influential group to suppress other groups.

Some small questions for the authors to consider would be:

1. Could more be said about the approximate intensity of the CDW peak compared to other cuprates? My experience is that cuprate CDW peaks tend to have roughly the same Q-integrated intensity, so they are mainly varying in correlation length.

2. Is there a special technical reason why Fig 3a was done via radial scans at different phi? This is a strange choice. A phi scan would arguably be more natural.

Reviewer #3 (Remarks to the Author):

I have read with attention the paper by Tam et al. which reports on the observation of a long-range CDW in overdoped curate, a result that I find both surprising and fascinating. To make a long story short, I think this is a very important paper, that contains a remarkable set of data (which establishes for good the ubiquity of the CDW now really seen in all the cuprates families), is nicely written and beside only a few remarks that need to be addressed, I strongly recommend to accept it for publication in Nature Communications.

i) I will start here with the most important point. The authors mention that in the methods section that the L component was set to half integer value in order to maximize the CDW intensity: I cannot

believe that the L-dependence of the signal has not been investigated. In my opinion this is the main qualitative difference between the long-range and short-range CDW in other cuprates. I think (but I am happy to be proven wrong) that except for YBCO there are no clear evidence for this doubling of the unit cell along the c-axis. In all other cases the correlations are extremely poor (so poor that correlation is probably a bad wording) and the CDW mostly 2D. In Hg1201, according to Tabis et al. PRB 96, 134510 (2017), the CDW signal is visible only at integer L and vanishes at half-integer L, due to the single layer structure according to these authors.
So I think it would be a fairly important issue to discuss here, for instance in view of the fact that the authors note that the electronic states involved in the CDW show little dispersion along kz.

Minor points:

ii) In the introduction it is mentioned that 'the CDW induces a sign reversal of the Hall number ... [12]'. Ref. 12 has been published before the first reports of CDW, so this statement is factually incorrect. The fact that there is a sign reversal in the Hall effect is not disputed here, but ref. 12 does not associate it explicitly with the CDW. Of course one is entitled to take a fresh look at these data building on the knowledge accumulated since their publication, but the sign change itself occurs at fairly low temperature and a high field is needed to observe it. In fact none of the characteristic temperatures associated with the sign changes in the Hall effect reported in ref. 12 can be unambiguously associated with the CDW formation. I am myself a strong supporter of the fact that the change of sign of nH and the CDW are associated, but this is neither claimed nor really supported by ref. 12.

iii) on page 2 the authors refer to non-resonant x-scattering and later on to non-resonant diffraction, which I find both confusing as this is usually associated with hard x-rays. I would rather mention that the incident energy of the x-ray beam has been 'detuned' from the main resonance.
In any event, the generic term scattering' should be preferred to 'diffraction', which generally refers to the special case where a constructive interference poccurs.

iv)On page 3 the authors mention that they believe that the peak seen at 160K in the Tc=56K sample is of spurious origin. I understand that this statement is made out of caution, but I would welcome a more open-minded approach. After all it does not look very different from the 10degrees signal seen in Fig. 3a, and there have been reports, in LBCO I believe, of a temperature dependent modulation wave vector. So yes, it may spurious, but I would indicate that further investigation are necessary there.

Charge density waves and Fermi-surface reconstruction in the clean overdoped cuprate superconductor $Tl_2Ba_2CuO_{6+\delta}$
Response to Reviewers Comments

We are pleased to see that all three Reviewers recommend publication of our manuscript in Nature Communications, once the issues they have raised have been addressed. We thank them for their insightful comments. Below we respond to each point in turn. Reviewer comments are copied, followed by our response and changes made to the manuscript. The changes to the new manuscript are shown in red. In addition, to the changes to the manuscript suggested by the reviewers we have also updated the section on Hall Effect and Fermi surface reconstruction to clarify the calculation and results.

REVIEWER #1

Comment 1:-The authors do not see the 60-meV phonon softening, which seems to happen in many other cuprates and has been associated with electron-phonon coupling. This is a crucially important clue here in the sense that if they do not see the softening, then the e-p coupling here might be weaker than in the underdoped case. Since this is so important, the authors should show how they extracted the phonon energy, and show more details of this analysis in the supplementary information. The resolution 48meV is comparable to that of other works, so the information of whether the phonon disperses is already in their data. It should be presented thoroughly in this paper, for both samples that show a CDW.

Reply 1: We agree with the reviewer that the soften is an important issue. The RIXS measurements had a strong elastic scattering present at all Q. This makes separating the phonons from the elastic scattering more difficult than in some other studies e.g. [1]. Because of beam-time constraints, only a limited number of spectra e.g. main text in Fig. 2 were counted for 17 minutes to obtain the best statistics. The spectra for the $p = 0.25$ and $p = 0.23$ compositions taken at $T \approx T_c$ together with the fit components and extracted phonon frequencies are presented in Supplementary Note 2. Within the uncertainty from fitting, our data does allow a weak softening for $|H|$ slightly larger than δ. However our data do not yield a strong conclusion on the magnitude of any softening and further work is required.

Changes 1: The discussion in the main text now states that the data allows a weak softening ($\lesssim$10%) for $H > \delta$. Phonon data and the fitting are included in Supplementary Note 2.

Comment 2: -Recently an inelastic intensity feature was observed across the whole qx-qy plane [2], and the authors have performed Q-RIXS maps across different azimuthal directions. In the current paper, the authors only show the elastic intensity as a function of azimuth, but they have the whole inelastic data to analyze. The authors should discuss if this inelastic feature seen in Bi2212 is observed in Tl2201.

Reply 2: In the work of Boschini *et al.* [2], an inelastic ring in the HK plane, with radius equal to the charge ordering wavevector was observed in the part of the RIXS spectrum above the region of collective low-energy excitations and below the dd excitations in Bi2212. This was interpreted as evidence of dynamical charge fluctuations. For the ζ scans we did at three azimuthal angles, we have checked to see whether this feature is present in our data. The integrated RIXS intensities scans in range $[500, 900]$ meV for the $p = 0.23$ composition are plotted in Supplementary Note 3. There is no obvious peak as observed by Boschini *et al.* [2].

Changes 2: We have added a comment stating that we do not observe the feature and show the supporting data in Supplementary Note 3.

REVIEWER #2

Comment 1: Could more be said about the approximate intensity of the CDW peak compared to other cuprates? My experience is that cuprate CDW peaks tend to have roughly the same Q-integrated intensity, so they are mainly varying in correlation length.

Reply 1: We have compared the Tl2201 data with LSCO ($p = 0.12$) data collected under similar conditions. The data and analysis are shown in Supplementary Note 4. We find that Tl2201 ($p = 0.23$) and LSCO ($p = 0.12$) have similar Q-integrated intensities, with Tl2201 ($p = 0.25$) having about half the Q-integrated intensity. This confirms the reviewer's expectations.

Changes 1: We have added a discussion on the Q integrated intensity in the main text with the data and analysis in Supplementary Note 4.

Comment 2: Is there a special technical reason why Fig 3a was done via radial scans at different phi? This is a strange choice. A phi scan would arguably be more natural.

Reply 2: We agree a phi scan may be more natural. We choose the 'ζ' scans to make sure that we passed through any (possibly) sharp peak/feature in H/ζ due to charge correlations or other origin. In the end, this type of scan was useful to address the comment 2 of reviewer # 1.

REVIEWER #3

Comment 1: The authors mention that in the methods section that the L component was set to half integer value in order to maximize the CDW intensity: I cannot believe that the L-dependence of the signal has not been investigated. In my opinion this is the main qualitative difference between the

long-range and short-range CDW in other cuprates. I think (but I am happy to be proven wrong) that except for YBCO there are no clear evidence for this doubling of the unit cell along the c-axis. In all other cases the correlations are extremely poor (so poor that correlation is probably a bad wording) and the CDW mostly 2D. In Hg1201, according to Tabis *et al.* [3], the CDW signal is visible only at integer L and vanishes at half-integer L, due to the single layer structure according to these authors. So I think it would be a fairly important issue to discuss here, for instance in view of the fact that the authors note that the electronic states involved in the CDW show little dispersion along kz.

Reply 1: We agree with the reviewer that this is an important issue in cuprates. The L-dependence of the CDW is believed to be determined by Coulomb interactions between the CDW in different layers and the interaction of the CDW and pinning sites [4, 5]. As the reviewer points out, the L-dependence of is different [6] for '3D' ($H = 1$) and '2D' ($L = 1/2$) CDW seen for example in YBCO, with stress/applied field or ambient conditions. In the present paper, we have measured at zero stress/field and believe that we are most likely to observe a '2D' CDW with short-range correlations along the c-axis. Empirically, we know that in zero field/zero stress the CDW is strongest at L=integer+1/2 in YBCO. In LSCO/LBCO, hard x-ray data (e.g. [7], Fig. 2) suggests this also strong at these positions L=integer+1/2. RIXS measurements on LSCO (e.g. [1]) performed close to half-integer positions observe a strong CDW signal i.e. they are consistent with the hard x-ray studies. As the reviewer points out, according to Tabis *et al.* [3] in Hg1201 the CDW is strongest at L=integer positions. We do not have a model to explain how the L modulation form, but note the three systems mentioned have three different structures: primitive tetragonal (P4/mmm) with bilayers (YBCO), primitive tetragonal without bilayers (Hg1201), and body centred tetragonal, I4/mmm (LSCO). Tl2201 has a body centred tetragonal lattice with CuO_6 octahedra and is most similar to LSCO. So we think it is likely have related CDW-Coulomb/impurity effects and $L = 2.5$ would be a reasonable choice.

In practice, the relatively large value of $\delta = 0.31$ and lattice parameter $c \approx 23.1$Å of Tl2201 determine the range of accessible values of L. We were able to work down to grazing incidence values of $\alpha \approx 9^\circ$, which allows $2 \lesssim L \lesssim 2.8$. H-scans near $L = 2$ would have a very large background because the small α. Choosing $L = 2.5$ allowed us to have a good H range. See Supplementary Note 1.

Changes 1: We have added supplementary Note 1 to explain the constraints on choosing L. We have added a sentence in the results section pointing to this note. In the discussion section, we discuss of the possible cause of $L = 1/2$ CDW peaks in cuprates to address the reviewer's point about the lack of dispersion along k_z.

Comment 2: In the introduction it is mentioned that 'the CDW induces a sign reversal of the Hall number ... [8]'. Ref. [8] has been published before the first reports of CDW, so this statement is factually incorrect. The fact that there is a sign reversal in the Hall effect is not disputed here, but ref. [8] does not associate it explicitly with the CDW. Of course one is entitled to take a fresh look at these data building on the knowledge accumulated since their publication, but the sign change itself occurs at fairly low temperature and a high field is needed to observe it. In fact none of the characteristic temperatures associated with the sign changes in the Hall effect reported in ref. [8] can be unambiguously associated with the CDW formation. I am myself a strong supporter of the fact that the change of sign of nH and the CDW are associated, but this is neither claimed nor really supported by ref. [8].

Reply 2: We agree the presentation in the first draft was not precise. We have rewritten the paragraph to be more accurate, separating the papers and noting what they found. (1) CDW order observed by [9, 10]; (2) FS reconstruction by "density wave order" with wavevector $(\delta, 0)$ predicted by [11, 12]; (3) Quantum oscillations measured [13]; (4) Hall number measured [8].

Changes 2: Paragraph 2, page 1, rewritten as described above.

Comment 3: On page 2 the authors refer to non-resonant x-scattering and later on to non-resonant diffraction, which I find both confusing as this is usually associated with hard x-rays. I would rather mention that the incident energy of the x-ray beam has been 'detuned' from the main resonance. In any event, the generic term scattering' should be preferred to 'diffraction', which generally refers to the special case where a constructive interference occurs.

Changes 3: Correct nomenclature regarding resonant and non-resonant scattering is now used throughout the main text.

Comment 4: On page 3 the authors mention that they believe that the peak seen at 160K in the Tc=56K sample is of spurious origin. I understand that this statement is made out of caution, but I would welcome a more open-minded approach. After all it does not look very different from the 10degrees signal seen in Fig. 3a, and there have been reports, in LBCO I believe, of a temperature dependent modulation wave vector. So yes, it may spurious, but I would indicate that further investigation are necessary there.

Changes 4 We agree, this could be evidence of a temperature dependent δ, which would warrant further investigation in a future experiment. The sentence on page 2 regarding this has been adjusted.

[1] J. Lin, H. Miao, D. Mazzone, G. Gu, A. Nag, A. Walters, M. García-Fernández, A. Barbour, J. Pelliciari, I. Jarrige, M. Oda, K. Kurosawa, N. Momono, K.-J. Zhou, V. Bisogni, X. Liu, and M. Dean, Strongly correlated charge density wave in $La_{2-x}Sr_xCuO_4$ evidenced by doping-dependent phonon anomaly, Phys. Rev. Lett. **124**, 207005 (2020).

[2] F. Boschini, M. Minola, R. Sutarto, E. Schierle, M. Bluschke, S. Das, Y. Yang, M. Michiardi, Y. C. Shao, X. Feng, S. Ono,

R. D. Zhong, J. A. Schneeloch, G. D. Gu, E. Weschke, F. He, Y. D. Chuang, B. Keimer, A. Damascelli, A. Frano, and E. H. da Silva Neto, Dynamic electron correlations with charge order wavelength along all directions in the copper oxide plane, Nat. Commun. **12**, 597 (2021).

[3] W. Tabis, B. Yu, I. Bialo, M. Bluschke, T. Kolodziej, A. Kozlowski, E. Blackburn, K. Sen, E. M. Forgan, M. v. Zimmermann, Y. Tang, E. Weschke, B. Vignolle, M. Hepting, H. Gretarsson, R. Sutarto, F. He, M. L. Tacon, N. Barišić, G. Yu, and M. Greven, Synchrotron x-ray scattering study of charge-density-wave order in $HgBa_2CuO_{4+\delta}$, Phys. Rev. B **96**, 134510 (2017).

[4] Y. Caplan and D. Orgad, Dimensional crossover of charge-density wave correlations in the cuprates, Phys. Rev. Lett. **119**, 107002 (2017).

[5] M. Hücker, Structural aspects of materials with static stripe order, Physica C: Superconductivity **481**, 3 (2012).

[6] J. Chang, E. Blackburn, O. Ivashko, A. T. Holmes, N. B. Christensen, M. Hücker, R. Liang, D. A. Bonn, W. N. Hardy, U. Rütt, M. v. Zimmermann, E. M. Forgan, and S. M. Hayden, Magnetic field controlled charge density wave coupling in underdoped $YBa_2Cu_3O_{6+x}$, Nat. Commun. **7**, 11494 (2016).

[7] T. P. Croft, C. Lester, M. S. Senn, A. Bombardi, and S. M. Hayden, Charge density wave fluctuations in $La_{2-x}Sr_xCuO_4$ and their competition with superconductivity, Phys. Rev. B **89**, 224513 (2014).

[8] D. LeBoeuf, N. Doiron-Leyraud, B. Vignolle, M. Sutherland, B. J. Ramshaw, J. Levallois, R. Daou, F. Laliberté, O. Cyr-Choinière, J. Chang, Y. J. Jo, L. Balicas, R. Liang, D. A. Bonn, W. N. Hardy, C. Proust, and L. Taillefer, Lifshitz critical point in the cuprate superconductor $YBa_2Cu_3O_y$ from high-field Hall effect measurements, Phys. Rev. B **83**, 054506 (2011).

[9] G. Ghiringhelli, M. L. Tacon, M. Minola, S. Blanco-Canosa, C. Mazzoli, N. B. Brookes, G. M. D. Luca, A. Frano, D. G. Hawthorn, F. He, T. Loew, M. M. Sala, D. C. Peets, M. Salluzzo, E. Schierle, R. Sutarto, G. A. Sawatzky, E. Weschke, B. Keimer, and L. Braicovich, Long-range incommensurate charge fluctuations in (Y,Nd)$Ba_2Cu_3O_{6+x}$, Science **337**, 821 (2012).

[10] J. Chang, E. Blackburn, A. T. Holmes, N. B. Christensen, J. Larsen, J. Mesot, R. Liang, D. A. Bonn, W. N. Hardy, A. Watenphul, M. v. Zimmermann, E. M. Forgan, and S. M. Hayden, Direct observation of competition between superconductivity and charge density wave order in $YBa_2Cu_3O_{6.67}$, Nat. Phys. **8**, 871 (2012).

[11] N. Harrison, Near doping-independent pocket area from an antinodal Fermi surface instability in underdoped high temperature superconductors, Phys. Rev. Lett. **107**, 186408 (2011).

[12] A. Allais, D. Chowdhury, and S. Sachdev, Connecting high-field quantum oscillations to zero-field electron spectral functions in the underdoped cuprates, Nat. Commun. **5**, 5771 (2014).

[13] D. LeBoeuf, N. Doiron-Leyraud, J. Levallois, R. Daou, J.-B. Bonnemaison, N. E. Hussey, L. Balicas, B. J. Ramshaw, R. Liang, D. A. Bonn, W. N. Hardy, S. Adachi, C. Proust, and L. Taillefer, Electron pockets in the Fermi surface of hole-doped high-T_c superconductors, Nature **450**, 533 (2007).

REVIEWERS' COMMENTS

Reviewer #1 (Remarks to the Author):

The authors have adequately addressed the questions I raised, as well as the questions raised by the other referees. This work is now ready for publication in Nature Communications, in my opinion. The verification of charge order in the overdoped regime of the cuprates is a crucially important piece of information.

Reviewer #2 (Remarks to the Author):

The authors have provided good-quality responses to all the questions and made some worthwhile edits to the text. I support the publication of this work in Nat. Comm. in this form.

Reviewer #3 (Remarks to the Author):

The authors have addressed adequately all my comments (as well as those of the other referees), and I am now happy to recommend the publication of this paper in Nature Communications.

REVIEWERS' COMMENTS

Reviewer #1 (Remarks to the Author):

The authors have adequately addressed the questions I raised, as well as the questions raised by the other referees. This work is now ready for publication in Nature Communications, in my opinion. The verification of charge order in the overdoped regime of the cuprates is a crucially important piece of information.

Reviewer #2 (Remarks to the Author):

The authors have provided good-quality responses to all the questions and made some worthwhile edits to the text. I support the publication of this work in Nat. Comm. in this form.

Reviewer #3 (Remarks to the Author):

The authors have addressed adequately all my comments (as well as those of the other referees), and I am now happy to recommend the publication of this paper in Nature Communications.

Our Response

No response required to the reviewers kind comments.
